# The Use of a Handheld Ultrasound Device to Guide the Axillary Vein Access during Pacemaker and Cardioverter-Defibrillator Implantation. A Feasibility Study

**DOI:** 10.31083/j.rcm2308258

**Published:** 2022-07-20

**Authors:** Biagio Sassone, Giuseppe Simeti, Santo Virzì, Giovanni Pasanisi, Daniele Muser

**Affiliations:** ^1^Department of Translational Medicine, University of Ferrara, 44121 Ferrara, Italy; ^2^Department of Emergency, Division of Cardiology, SS.ma Annunziata Hospital, Azienda Unità Sanitaria Locale di Ferrara, 44042 Cento, Ferrara, Italy; ^3^Department of Emergency, Division of Cardiology, Delta Hospital, Azienda Unità Sanitaria Locale di Ferrara, 44023 Lagosanto, Ferrara, Italy; ^4^Cardiothoracic Department, Udine Civil Hospital, 33100 Udine, Italy

**Keywords:** ultrasound, handheld, axillary vein, pacemaker, cardioverter-defibrillator

## Abstract

**Background:**

Although ultrasound guidance for axillary vein (AV) access (USGAVA) has been described as a reliable technique for cardiac implantable electronic device (CIED) implantation, no data is available on the use of handheld ultrasound devices (HUD) in such a setting.

**Objective:**

We investigated the feasibility of using a HUD for USGAVA in patients referred to our Institution for CIED implantation.

**Methods:**

The procedure details of 80 consecutive patients undergoing USGAVA (Group-1) from June 2020 to June 2021 were prospectively collected and compared to those of an age and sex-matched cohort of 91 patients (Group-2) who had undergone AV access with the traditional venipuncture guided by fluoroscopic landmarks.

**Results:**

The two groups were comparable for the success rate of venous access (92.5% versus 93.4%, *p* = 0.82), complication rate (1.3% versus 0.9%, *p* = 1.0), and procedure time (71 ± 32 min versus 70 ± 29 min, *p* = 0.9). However, Group-2 had a longer X-ray exposure time (7.6 ± 8.4 min versus 5.7 ± 7.3 min, *p* = 0.03). In Group-1, the univariate logistic regression analysis demonstrated that the AV diameter was associated with successful USGAVA (odds ratio = 3.34, 95% confidence interval 1.47–7.59, *p *< 0.01), with a 3-fold increase of probability of success per each 1 mm increase in the AV diameter.

**Conclusions:**

USGAVA using a HUD for CIED implantation is a feasible, effective, and safe technique; moreover, it saves X-ray exposure time without lengthening the implant procedure time.

## 1. Introduction

Since the 1960s, the implantation of cardiac implantable electronic devices (CIEDs) through endovascular routes, including pacemakers, cardioverter-defibrillators, and devices for cardiac resynchronization therapy (CRT), has rapidly gained widespread acceptance by operators. In 2013, a European Heart Rhythm Association survey showed that cephalic vein dissection and subclavian vein puncture are the preferred venous access for CIED lead insertion [1]. While being very safe in preventing severe complications usually caused by traditional puncture of the subclavian or axillary vein, the cephalic vein has a lower success rate and longer procedural time [2]. Conversely, subclavian vein access is highly effective, quick to perform, and can accommodate multiple leads because of its large size [3]. Nevertheless, the approach to the subclavian vein, requiring venous access through an intrathoracic puncture, has feared complications that, although uncommon, may be potentially life-threatening, such as haemothorax or tensive pneumothorax and lead crush [4, 5].

Over the last years, axillary vein (AV) access has provided an alternative technique safer and more successful than the subclavian vein and cephalic vein, respectively [2, 6]. The standard approach to the AV is performed by an extrathoracic venipuncture under fluoroscopy guidance, with or without contrast venography [7]. However, ionizing radiation exposure to both operators and patients, as well as the risk of iodinated contrast medium allergy and nephropathy are non-negligible disadvantages of such a method. Nevertheless, the use of ultrasound as real-time guidance to puncture the AV may overcome these limits, as first described in the late ‘90s’ [8]. A growing amount of literature indicates ultrasound-guided AV access (USGAVA) as a safe, effective, and time-saving alternative to other traditional techniques for device implantation while avoiding x-ray exposure and contrast medium use [9, 10]. However, although the United States Agency for Healthcare Research and Quality has strongly recommended ultrasound guidance for central venous access [11], USGAVA has yet to achieve widespread acceptance from operators. Maneuvering bulky ultrasound machines near the operating field and the need for a second operator are perceived by operators as an impediment to the workflow. In the last few years, the progressive miniaturization of ultrasound machines with device sizes comparable to current smartphones may facilitate the spread of the technique.

This study aimed to assess the feasibility, efficacy, and safety of USGAVA performed as a single-operator maneuver using a pocket-sized handheld ultrasound device (HUD) in patients undergoing CIED implantation.

## 2. Materials and Methods

### 2.1 Patient Selection 

This study enrolled, from June 2020 to June 2021, all consecutive adult (>18 years) patients (Group-1) referred to our tertiary cardiology center for pacemaker, cardioverter-defibrillator, and CRT device implantation who systematically underwent USGAVA using a HUD as the initial approach for leads insertion into the central venous system through the AV. Device upgrades were excluded from the study. USGAVA was performed by two operators with skills and long-standing experience in ultrasound-guided venous access in electrophysiology. Written informed consent was obtained from all eligible patients before the implantation procedure.

### 2.2 Ultrasound-Guided AV Access Technique

**Fig. 1.**
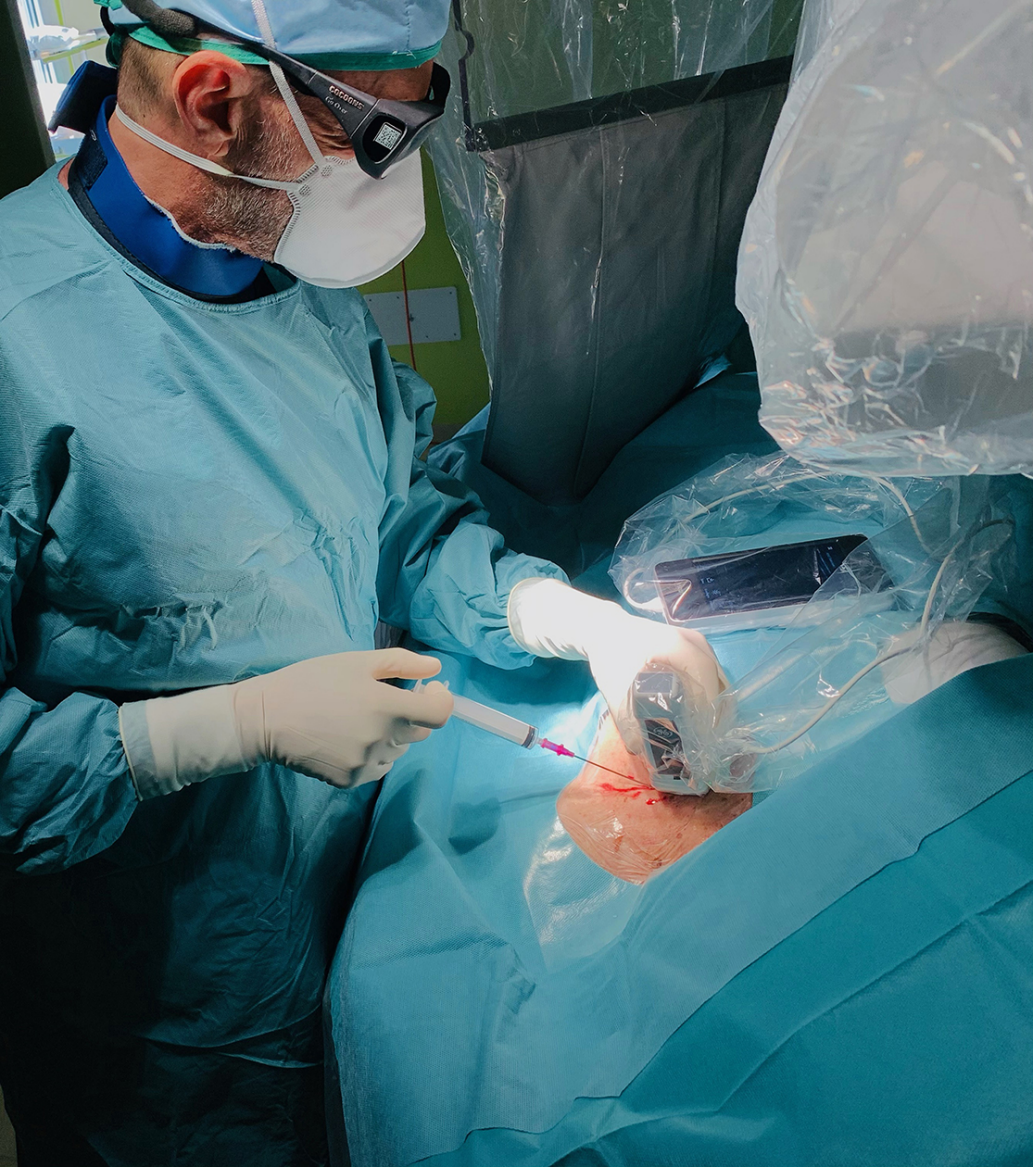
**Operating field arrangement during a pacemaker implantation procedure**. The entire ultrasound system is covered in a single sterile, transparent plastic sheath. The operator is carrying out the axillary vein puncture with ultrasound-guided freehand technique orienting the probe marker cranially. The needle is kept aligned to the probe’s centerline marker while imaging the longitudinal axis of the vessel.

In our practice, USGAVA is a single operator maneuver with the non-dominant hand holding the transducer and the dominant hand performing the venipuncture (Fig. 1). USGAVA is performed with Vscan Extend™ (GE Healthcare, Waukesha, WI, USA), a handheld ultrasound system with a high-frequency 3.3–8 MHz linear array transducer. Both the display unit and probe are covered in a single sterile, transparent plastic sheath with sterile gel applied directly over the probe inside the sheath. The skin is cleaned with 2% chlorhexidine solution for antisepsis at the infraclavicular area, and a sterile disposable surgical whole-body shaped drape with a preformed hole is applied to delimit the operation area. Thanks to its light weight (321 gr) and small size (168 × 76 × 22 mm), the display unit is easily positioned over the operating field, on the precordial zone of the patient’s chest, thus enabling the operator to view the real-time images while scanning the AV and the surrounding anatomical structures. Intravenous fluids are routinely administrated to increase the AV diameter and counteract its collapse. We run a saline infusion wide open through a peripheral vein of the ipsilateral arm for a few minutes before beginning ultrasound scanning until the introduction of the guidewires through the vein. For the same purpose, the operator might decide to place the patient in the Trendelenburg position, tilting the operating table head-down by 15°, especially in the absence of venous access for the saline infusion. The AV is easily identified and distinguished from the adjacent axillary artery based on the following characteristics: more superficial position, compressibility during gentle pressure over the skin with the footprint of the probe, lack of pulsation, dynamic inspiratory collapse, and visualization of the typical angled entering of the cephalic vein. Local anaesthesia with lidocaine hydrochloride 2% is performed along the planned needle trajectory, halting immediately before entering the AV. To impede compromising the ultrasound image quality, we take care to avoid micro air bubbles entering the tissues surrounding the vein when injecting the local anaesthesia. The puncture is performed before skin incision, with the freehand technique (i.e., without the aid of needle guides). We prefer to access the vein before pocket creation because, first, maneuvering the probe inside the pocket makes the maneuver more complex for the operator, and, secondly, micro air bubbles may enter the tissues and interfere with the image quality.

While advancing an 18-gauge needle, the ultrasound transducer is tilted to image the AV in the longitudinal axis, and the needle is kept aligned to the probe’s centerline marker and in-plane of the ultrasound beam to visualize the needle tip until tenting the vessel wall. Then, when the needle tip-induced vessel indent is evident, the needle is advanced by performing short jabs until entering the lumen, as confirmed by aspiration of venous blood. A 0.035” j-tip guidewire is then inserted through the needle into the vein and advanced to the inferior vena cava under fluoroscopic guide. For CIEDs requiring more than a single lead, additional venous accesses are obtained with multiple punctures (one puncture per lead) by moving the puncture site by 0.5 cm proximally along the AV run. Alternatively, a single-puncture approach with a retained guidewire could be used for multiple lead implantation, based on the operator’s preference. A linear skin incision is made with a #11 surgical scalpel medially to the guidewires. Then, the device pocket is created by a manual detachment of subcutaneous tissue planes above the pectoralis major muscle fascia and the guidewires are reached using blunt dissecting scissors through the subcutaneous tissue and drawn under the skin into the device pocket. Finally, a peel-away dilator/introducer assembly is inserted over the guidewire into the central venous system and the leads are implanted following the standard fashion. If USGAVA fails, a skin incision is made, a device pocket prepared and an alternative venous approach without ultrasound guidance, based on the operator’s preference, was attempted using the standard technique as described below.

### 2.3 Comparison Group 

Group-1 was compared with a historical group represented by all patients who underwent conventional procedures—without ultrasound guidance—for transvenous CIED implantation at our Institution, from June 2019 to May 2020 (Group-2). In these patients, fluoroscopy-guided AV puncture, either with or without contrast-venography, was used following the consolidated standard techniques for CIEDs implantation. Patients who underwent cephalic access were excluded from the analysis. The AV puncture is routinely performed after making the skin incision and creating the device pocket; and contrast-venography is used only when the venipuncture attempt guided by the fluoroscopic landmarks fails.

### 2.4 Management of Antithrombotic Therapy

As standard practice, in elective settings, oral anticoagulant therapy with vitamin K antagonists is not interrupted, targeting the international normalized ratio (INR) value between 2.0 and 3.0, preferably 2.0–2.5, on the procedure day. The CIED implantation is usually postponed if the INR value is over 3.0. Periprocedural bridging with either unfractionated heparin or low-molecular-weight heparin is not practiced at our Institution, but it might be considered for selected patients at very high risk of thromboembolism. Timing for both the holding and resumption of direct oral anticoagulants is based on the patient’s renal function according to the recommendations of the European Heart Rhythm Association, Heart Rhythm Society, and Asia Pacific Heart Rhythm Society [12]. Periprocedural either single or dual antiplatelet therapy is not interrupted.

### 2.5 Variables and Definitions

**Fig. 2.**
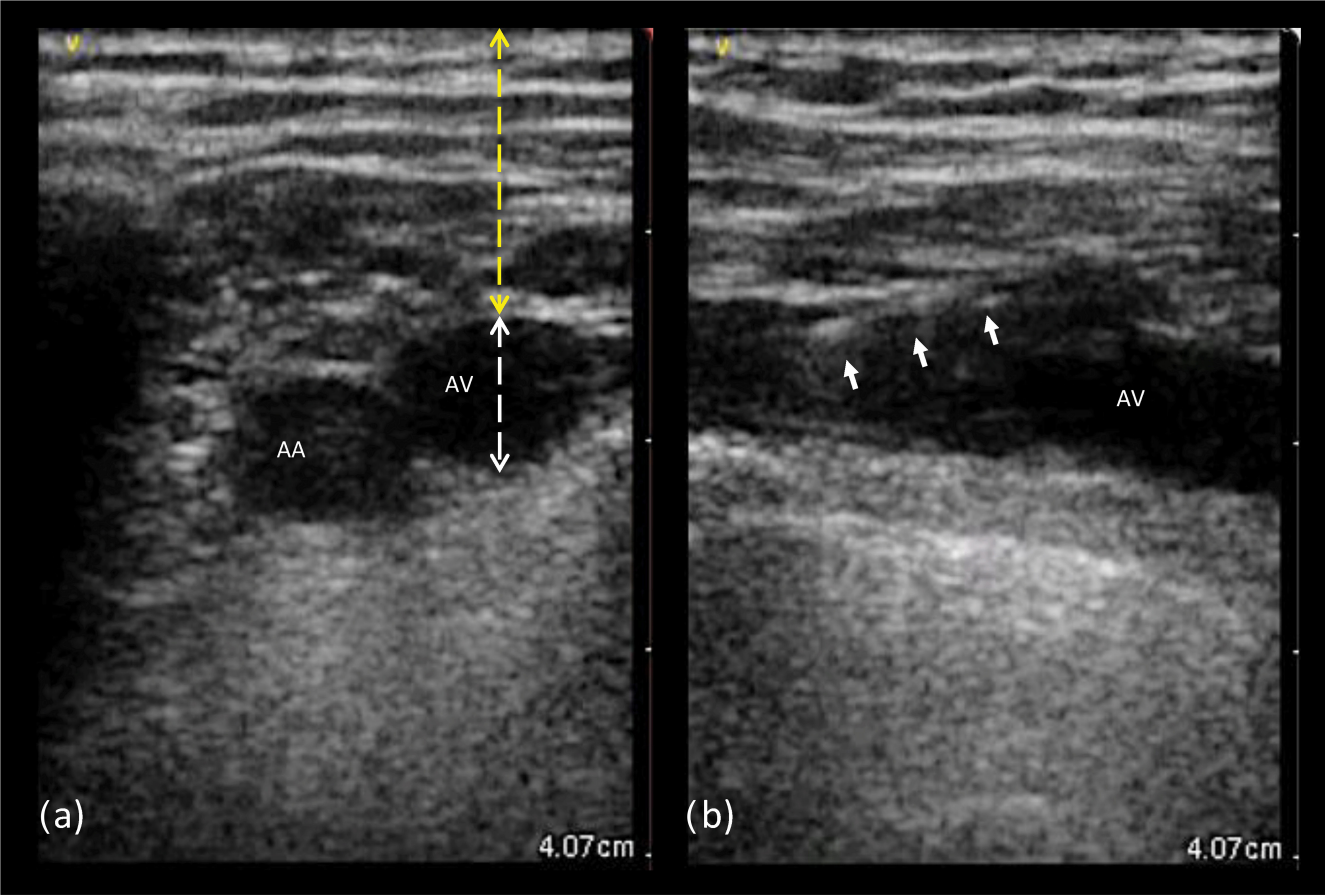
**Ultrasound images from left axillary vein**. (a) Transverse view: the probe is aligned perpendicular to the axis of both the axillary artery and vein, which appear as anechoic circular images; the anatomic landmarks to measure the axillary vein depth (yellow) and diameter (white) are shown. (b) Longitudinal view: the probe is aligned with the axillary vein course, creating a tubular image of the vessel; the white arrows indicate the guidewire entering the target vein lumen. AA, axillary artery; AV, axillary vein.

Baseline demographics, biometric and clinical characteristics, procedural details, and complications occurring within 30 days after the procedure were collected prospectively in Group-1, either as continuous or categorical variables. The same parameters were retrospectively gathered from Group-2, reviewing patient electronic medical records and case-procedure logs via the official regional web-based registry RERAI (Registry of Emilia Romagna on Arrhythmia Interventions) for comparative data. In Group-1, ultrasound images of the AV were acquired by 4-second video clips and stored in the HUD. The captured images were then manually reviewed immediately after the end of the procedure to measure the vein depth and the maximum vein diameter (Fig. 2). To evaluate the reproducibility of the axillary vein measurements, a reliability analysis was performed as follows: the vein diameter was remeasured by the same operator in all 80 patients undergoing USGAVA for assessment of intraobserver variability; then, a second blinded operator measured the vein diameter in a group of 20 out of 80 patients to assess the interobserver variability. The overall procedural duration was measured from the performance of local anaesthesia to the completion of the skin suture. A venous access attempt was considered successful when all the leads to be implanted were imaged inside the inferior vena cava under fluoroscopy. In Group-1, if any other technique other than USGAVA was used, even for only one lead in the case of devices with multiple leads, the procedure was labelled as unsuccessful. In Group-2, when the operator had decided to change the initial chosen approach to gain central venous access with another strategy, including the additional use of contrast-venography, the procedure was considered unsuccessful. A chest X-ray was systematically performed the day after the procedure, with the patient standing whenever possible, to check the occurrence of either pneumothorax or lead dislodgement. The wound at the skin incision site was checked daily during the hospital stay and, subsequently, on removing the skin stitches, usually at day 12 from implantation.

### 2.6 Study Endpoints

The study endpoints were procedural-related outcomes including successful venous access, total procedure time, and total X-ray exposure time. Also, we collected the complications occurring within 30 days from CIED implantation including pneumothorax, pocket haematoma requiring any intervention (e.g., halting antithrombotic therapy, drainage positioning, etc.), infection, venous thrombosis of the accessed vein, or other procedural-related complications.

### 2.7 Statistical Analysis 

Statistical analyses were performed using IBM SPSS version 26.0 software (SPSS Inc, Chicago, IL, USA). Continuous variables are expressed as means ± standard deviations if normally distributed or medians (25th–75th percentile) if not normally distributed. All continuous variables were tested for normal distribution using the 1-sample Kolmogorov–Smirnov test. Categorical data are expressed as counts and percentages. Continuous variables were compared using independent-sample parametric (unpaired Student *t*) or non-parametric (Mann-Whitney U) tests. Categorical variables were compared using the Chi-Square test or Fisher Exact test when appropriate. Univariate logistic regression analysis was performed to evaluate the association between successful venous access and baseline covariates. The following variables potentially affecting a successful USGAVA were entered in the univariate model: age (per year), male gender, body mass index (per Kg/$m^{2}$), body surface area (per $m^{2}$), left ventricular ejection fraction (per %), diabetes mellitus, coronary artery disease, chronic obstructive pulmonary disease, hypertension, history of cardiac surgery, creatinine (per mg/dL), axillary vein depth (per mm), and axillary vein diameter (per mm). Receiver operating characteristic (ROC) curve analysis and the calculation of the area under the curve (AUC) were used to determine the discriminatory power and the optimal cut-off value of the AV diameter in predicting successful venous access. For the analysis of intraobserver and interobserver AV measurement variability, we used the intraclass correlation coefficient (ICC). Two-tailed tests were considered statistically significant at the 0.05 level.

## 3. Results 

The study involved 171 participants, including 80 patients (Group-1) who underwent CIED implantation with USGAVA as the first-choice strategy for lead insertion. Baseline characteristics of the overall study population are listed in Table 1. The mean patient age was 78 ± 10 years, and 119 (70%) were males. Baseline demographics, biometric and clinical characteristics were comparable between Group-1 and Group-2.

**Table 1.** **Demographic and clinical characteristics of the study patients (n = 171)**.

Variable		USGAVA	Standard access	*p*-value
		(Group-1, n = 80)	(Group-2, n = 91)	
Age, years (±SD)		77 ± 10	78 ± 11	0.20
Male gender, n (%)		57 (71)	62 (68)	0.66
Body mass index, Kg/$m^{2}$ (±SD)		27.2 ± 4.5	28.9 ± 9.0	0.44
Body surface area, $m^{2}$ (±SD)		1.87 ± 0.16	1.84 ± 0.21	0.25
Success rate of the initial strategy for venous access, n (%)		74/80 (92.5)	85/91 (93.4)	0.82
Use of contrast venography, n (%)		1 (1)	6 (7)	0.12
Device type, n (%)				
	Single-chamber pacemaker	14 (17)	20 (22)	0.57
	Dual-chamber pacemaker	35 (44)	39 (43)	1.00
	Biventricular pacemaker	15 (19)	12 (13)	0.40
	Single-chamber cardioverter-defibrillator	9 (11)	15 (16)	0.38
	Dual-chamber cardioverter-defibrillator	0 (0)	0 (0)	-
	Biventricular cardioverter-defibrillator	7 (9)	5 (6)	0.55
Average number of leads per patient, n (±SD)		1.9 ± 0.69	1.7 ± 0.66	0.08
Right sided implantation site, n (%)		2 (2.5)	0 (0)	0.23
Left ventricular ejection fraction, % (±SD)		50.2 ± 15.1	51.1 ± 15.1	0.64
Diabetes mellitus, n (%)		19 (24)	26 (29)	0.49
Coronary artery disease, n (%)		24 (30)	33 (36)	0.42
Chronic obstructive pulmonary disease, n (%)		11 (14)	16 (18)	0.53
Hypertension, n (%)		59 (74)	60 (66)	0.32
History of cardiac surgery, n (%)		10 (13)	10 (11)	0.81
Creatinine, mg/dL (±SD)		1.01 ± 0.39	1.14 ± 0.78	0.13
Antithrombotic therapy, n (%)		62 (78)	71 (78)	1.00
	Vitamin K antagonists	14 (18)	14 (15)	0.84
	Direct oral anticoagulants	24 (30)	17 (19)	0.11
	Single antiplatelet therapy	18 (23)	27 (30)	0.30
	Dual antiplatelet therapy	2 (3)	6 (7)	0.29
	Anticoagulant plus single antiplatelet therapy	2 (3)	6 (7)	0.29
	Anticoagulant plus dual antiplatelet therapy	2 (3)	1 (1)	0.60
Total procedure duration time, min (±SD)		71 ± 32	70 ± 29	0.90
Total fluoroscopy exposure time, min (±SD)		5.7 ± 7.3	7.6 ± 8.4	0.03
Complications, n (%)		1 (1.3)	1 (0.95)	1.00

Both the success rate of the initial strategy for venous access and the 30-day complication rate showed no difference between the study groups. In Group-1, successful USGAVA was obtained in 74 of 80 patients (92.5%), resulting in a total of 142 out of 152 leads implanted (94%). A single-puncture approach with a retained guidewire for the insertion of multiple leads was used in 4 patients who received a dual-chamber pacemaker. In two other patients, the operator, after an unsuccessful first attempt with a standard 0.035” j-tip guidewire, used a thinner 0.028” guidewire for advancing through the AV. In the remaining patients, USGAVA followed the standard technique as described above. In 6 patients (7.5%) USGAVA was unsuccessful as the operator failed to enter the lumen of the vein with the needle tip. All these patients underwent successful CIED implantation via AV cannulation by conventional fluoroscopy-guided approach, with (1 patient) or without (5 patients) contrast-venography. Following our definition, complications occurred in only one patient who had an upper extremity deep venous thrombosis, ipsilateral to the implantation site, that was diagnosed 2 weeks after dual-chamber pacemaker implantation with successful USGAVA, while taking single antiplatelet therapy.

In Group-2, venous access was successful in 85 out of 91 patients (93.4%). In the remaining 6 patients, the operator, after failing the venous cannulation with the initial strategy, decided to use contrast venography for guiding the AV puncture. The complications included one haemothorax in a patient undergoing cardioverter-defibrillator implantation on dual antiplatelet therapy, which was successfully managed with a chest tube insertion.

While the overall procedure time did not differ between the two study groups, the total fluoroscopy exposure time during the procedure was significantly higher in Group-2 compared to Group-1 (7.6 ± 8.4 min versus 5.7 ± 7.3 min, *p* = 0.03).

To investigate predictors of failed ultrasound-guided AV cannulation for CIED implantation in our study population, we compared the measured variables between Group-1 patients with successful and unsuccessful USGAVA (Table 2). Patients with a failed attempt of USGAVA exhibited a trend towards a higher body mass index (30.1 ± 6.6 Kg/$m^{2}$ versus 26.9 ± 4.2 Kg/$m^{2}$, *p* = 0.07) and a significantly smaller AV diameter (1.8 ± 1.8 mm versus 9.2 ± 3.3 mm, *p *< 0.01) compared to successful USGAVA patients. None of the other compared variables was statistically significant. The univariate logistic regression analysis demonstrated that the AV diameter was significantly associated with USGAVA success (odds ratio = 3.34, 95% CI 1.47 to 7.59, *p *< 0.01), with a 3-fold increase of probability of success per each 1 mm increase in the AV diameter. Other variables were not correlated with USGAVA success. Then, at the ROC curve analysis, a cut-off value for AV diameter of ≥5.7 mm showed the highest accuracy (sensitivity 84%, and specificity 100%) to predict successful ultrasound-guided venous puncture (AUC = 0.97, *p *< 0.001). The intra- and interobserver agreement for AV diameter measurement was excellent: ICC 0.98 (95% CI 0.98–0.99, *p *< 0.001) and 0.98 (95% CI 0.96–0.99, *p *< 0.001), respectively.

**Table 2.** **Comparison between patients with successful and unsuccessful ultrasound-guided axillary vein access**.

Variable		Successful USGAVA	Unsuccessful USGAVA	*p*-value
		(n = 74)	(n = 6)	
Age, years (±SD)		77.3 ± 8.8	70.8 ± 15.5	0.23
Male gender, n (%)		52 (70)	5 (83)	0.50
Body mass index, Kg/$m^{2}$ (±SD)		26.9 ± 4.2	30.1 ± 6.6	0.07
Body surface area, $m^{2}$ (±SD)		1.87 ± 0.15	1.94 ± 0.28	0.60
Device type, n (%)				
	Single-chamber pacemaker	13(18)	1 (17)	0.96
	Dual-chamber pacemaker	32 (43)	3 (50)	0.75
	Biventricular pacemaker	15 (20)	0 (0)	1.00
	Single-chamber cardioverter-defibrillator	7 (9)	2 (33)	0.08
	Dual-chamber cardioverter-defibrillator	0 (0)	0 (0)	-
	Biventricular cardioverter-defibrillator	7 (10)	0 (0)	0.43
Average number of leads per patient, n (±SD)		1.9 ± 0.68	1.7 ± 0.52	0.40
Right sided implantation site, n (%)		2 (2.5)	0	0.68
Left ventricular ejection fraction, % (±SD)		50.6 ± 14.8	45.7 ± 19.6	
Diabetes mellitus, n (%)		17 (23)	2 (33)	0.57
Coronary artery disease, n (%)		19 (26)	2 (33)	0.68
Chronic obstructive pulmonary disease, n (%)		10 (14)	1 (17)	0.83
Hypertension, n (%)		54 (76)	5 (83)	0.58
History of cardiac surgery, n (%)		10 (14)	0 (0)	1.00
Creatinine, mg/dL (±SD)		1.02 ± 0.40	0.81 ± 0.15	0.17
Antithrombotic therapy, n (%)				
	Vitamin K antagonists	13 (18)	1 (17)	0.83
	Direct oral anticoagulants	23 (31)	1 (17)	0.66
	Single antiplatelet therapy	17 (23)	1 (17)	0.62
	Dual antiplatelet therapy	1 (4)	1 (17)	0.27
	Anticoagulant plus single antiplatelet therapy	2 (3)	0 (0)	1.00
	Anticoagulant plus dual antiplatelet therapy	2 (3)	0 (0)	1.00
Total procedure duration time, min (±SD)		71 ± 33	69 ± 14	0.66
Total fluoroscopy exposure time, min (±SD)		5.6 ± 7.5	6.1 ± 4.9	0.55
Complications, n (%)		1 (1.3)	0 (0)	1.00
Axillary vein depth, mm (±SD)		22.2 ± 6.8	20.6 ± 6.1	0.55
Axillary vein diameter, mm (±SD)		9.2 ± 3.3	1.8 ± 1.8	<0.01

## 4. Discussion

To the best of our knowledge, this is the first report on the use of a pocket-sized handheld ultrasound system for real-time image-guided vascular access during transvenous CIED implantation. The results of our study enrich the growing amount of data indicating ultrasound-guided access of the AV for pacemaker or cardioverter-defibrillator leads implantation as a feasible and comparable alternative technique to the traditionally used AV puncture guided by fluoroscopy landmarks.

### 4.1 Main Results 

In our study, USGAVA, while requiring less x-ray exposure (5.7 ± 7.3 min versus 7.6 ± 8.4 min, *p* = 0.03), resulted in a similar total procedure time (71 ± 32 min versus 70 ± 29 min, *p* = 0.9), efficacy (success rate, 92.5% versus 93.4%, *p* = 0.8), and safety (complication rate, 1.3% versus 0.95%, *p* = 1.0) compared to the standard venous access via fluoroscopy. The overall complication rate was low, including an upper extremity deep venous thrombosis and a haemothorax in the USGAVA and fluoroscopy-guided group, respectively. Haemothorax is an infrequent insertion-related complication caused by an accidental axillary artery puncture. The use of ultrasound to guide the AV puncture may have prevented such complications by direct visualization of the needle tip, thus avoiding injuries to the vessel.

### 4.2 USGAVA versus Cephalic Vein Cutdown

Like most of the literature concerning the performance of USGAVA, our results refer to a comparison with fluoroscopy-guided venipuncture techniques. We excluded from the analysis patients undergoing cephalic vein access because it is rarely used at our Institution. A recent multicenter randomized clinical trial indicated USGAVA as superior in terms of success rate (97.7% versus 54.5%; *p *< 0.001) with a similar complication rate (2.3% versus 11.4%; *p* = 0.20), compared to cephalic vein cut-down in pacemaker and cardioverter-defibrillator implantation [9]. However, we acknowledge that in operators with skilled hands, cephalic vein cutdown has an excellent success rate, with the potential to accommodate placement of multiple leads with more than 90% success in delivering cardiac resynchronization therapy [13].

### 4.3 Comparison with Other Recent USGAVA Experiences 

As shown in Table 3 (Ref. [9, 10, 14, 15, 16, 17, 18, 19]), our results paralleled the most recent literature on ultrasound-guided AV access for the same procedure type. Indeed, although the studies were somewhat different in ultrasound-guided venipuncture techniques and ultrasound system machines (i.e., on-cart, portable, or wireless), the success rate proved similar across the patient groups with USGAVA, including our procedures performed by a handheld device [9, 10, 14, 15, 16, 17, 18, 19]. Conversely, the complication rate was quite different between studies, probably affected either by the different follow-up duration or the broad definition of procedure-related complications, as some of these were unlikely related to the venous cannulation technique. However, in our study, the high percentage of patients on antithrombotic therapy (78%) highlights the safety of the technique, especially with regard to the risk for hematoma. Similarly, ElJamili and coll. showed no hematoma in 180 patients under antithrombotic therapy undergoing CIED implantation with USGAVA, including cardioverter-defibrillators and CRT [17].

**Table 3.** **Descriptive comparison between our study and other recent published experiences on ultrasound-guided axillary vein access in cardiac implantable electronic devices implantation**.

		Our data	Esmaiel [14]	Franco [15]	Lin [16]	Liccardo [10]	Tagliari [9]	Eljamili [17]	De Sensi [18]	Chandler [19]
Study design		Single center, prospective, observational	Single center, retrospective	Single center, prospective, observational	Single center, retrospective	Single center, randomized	Multicenter, randomized	Multicenter, prospective, observational	Single center, retrospective	Single center, retrospective
Number of patients		80	403	50	137	116	44	200	119	187
Mean age, year ± SD		77 ± 10	N/A	74 ± 11	68 ± 14	74 ± 13	67.5 ${\mathrm{(55–76)}}^{a}$	78 ± 10	79 ± 9	69 ± 13
Male gender, %		71	N/A	56	63	57	59	58	63	67
Number of leads		142	648	86		207	75	360	204	396
Ultrasound system machine		handheld ultrasound device	on-cart ultrasound machine	wireless ultrasound transducer	portable laptop ultrasound systems	on-cart ultrasound machine	on-cart ultrasound machine	portable laptop ultrasound systems	on-cart ultrasound machine	wireless ultrasound transducer
Ultrasound section to image axillary vein		longitudinal	transverse	transverse	transverse	transverse	longitudinal/transverse	transverse	longitudinal	longitudinal
Ultrasound-guided venipuncture		before skin incision	after skin incision, inside the device pocket	before skin incision	before skin incision	before skin incision	before skin incision	before skin incision	before skin incision	before skin incision
Success rate for USGAVA, %		92.5	99.3	98	100	91	97.7	91	95	95
Device types, n (%)										
	Pacemaker	49 (61)	403 (100)	36 (72)	N/A	46 (40)	29 (66)	134 (67)	93 (78)	75 (40)
	Cardioverter-defibrillator	9 (11)	0	10 (22)	N/A	70 (60)	15 (34)	12 (6)	13 (11)	69 (37)
	CRT	22 (28)	0	4 (8)	27(20)	0	0	34 (27)	12 (11)	43 (23)
	Upgrade	0	0	0	N/A	0	0	14 (7)	4 (3)	8 (4)
Total fluoroscopy time, min		6.1 ± 7.3	N/A	N/A	N/A	N/A	N/A	8.5 ± 10.7	N/A	3.6 ${\mathrm{(2.0–5.5)}}^{a}$
Anticoagulation therapy at implant, %		53	N/A	42	N/A	48	N/A	52	34	42
Single antiplatelet therapy at implant, %		25	N/A	24	N/A	N/A	N/A	37	35	71
Combined antithrombotic therapy at implant, %		10	N/A	0	N/A	N/A	N/A	9	N/A	N/A
Complications, n (%)		1 (1.3)	2 (0.5)	1 ${\mathrm{(2)}}^{b}$	3 (2.2%)	4 ${\mathrm{(3)}}^{b}$	1 (2.3)	0 (0)	4 (3.4)	7 ${\mathrm{(4)}}^{c}$

Comparison refers to patient groups approached by ultrasound guided technique; combined antithrombotic therapy at implant means dual antiplatelet therapy, dual antithrombotic therapy, or triple antithrombotic therapy. ^a^ median value (Q1–Q3); ^b^ observation time for complication occurrence longer than 30 days; ^c^ broader definition of pocket hematoma.

### 4.4 Handheld Ultrasound Devices versus Standard Ultrasound Systems 

All published studies on USGAVA in CIED implantation used, initially, stationary high-end ultrasound machines and, more recently, mobile on-cart or portable laptop ultrasound systems. The manual handling of such bulky ultrasound systems in the operating room, the transducer wires over the surgical field, and the need for an additional operator at the console for tuning the echo imaging while the primary operator is attempting the venipuncture might endanger the maintenance of the sterility and hinder the procedure workflow. These conditions likely contributed to hampering the spread of USGAVA in clinical practice. Recently, Franco and coll. showed that USGAVA, performed with a portable laptop ultrasound system with a wireless transducer, proved highly effective and safe in CIED implantation [15]. As underlined by the authors, not having to deal with transducer wires over the operating field and the possibility to tune the image from the probe by the same operator who is attempting the venipuncture (without the need for a second operator) both represented advantages compared to the traditional ultrasound systems.

### 4.5 Feasibility of Using a Handheld Ultrasound Device in CIED Implantation

In the last years, technological improvements have engendered a progressive miniaturization of ultrasound machines, with device sizes comparable to current smartphones. In 2019, a position statement of the European Association of Cardiovascular Imaging highlighted the potentials of using HUD in different clinical settings, including vascular invasive procedures such as central venous catheter insertion [20]. A randomized study of performance on a simulation model showed that the imaging qualities were similar between pocket-sized and standard ultrasound devices to guide internal jugular venipuncture [21]. Recently, in a prospective randomized clinical trial by Yamamoto and coll., the use of a HUD for internal jugular venipuncture proved not inferior to a standard ultrasound on-cart system, despite differences in visibility because of the lower device performance of the pocket-sized devices [22]. In our experience, despite the inherent technological limitations and restricted functions of a pocket-sized device, the use of a HUD to guide vascular access during CIED implantation proved comparable in efficacy and safety to standard ultrasound systems with higher technological capabilities used in previous studies (Table 3). Given the small size and handiness of the ultrasound system, performing the USGAVA procedure with HUD placed over the operating field did not impede the efficacy of the maneuver, resulting in feasibility in 74 out of 80 patients. Finally, technical limits, including image resolution and a small screen, go along with miniaturized portable devices. In our experience, such technical issues did not negatively impact the operators’ performance for AV puncture. However, most of the currently available HUBs, including the one used in our study, allow the display to be mirrored onto a larger wireless monitor nearby, such as the screen for fluoroscopy. This capability could be helpful to overcome some technical limits related to the device’s small size.

### 4.6 Economic Issues 

Seto and coll. estimated USGAVA-related additional professional reimbursement costs similar to venography, although with higher technical fees [23]. At our Institution, we roughly estimated an additional cost associated with USGAVA of €1.8/procedure comprising the sterile plastic sleeve (€0.8/unit) and disposal gel (€1/unit). The initial cost of the HUD with dual probe (phased-array and linear) should also be considered in the final estimate if not yet available in the operating room. Of note, the cost of the entire HUD is approximate to the sole cost of the vascular probe of high-end, portable on-cart or laptop ultrasound systems.

### 4.7 Predictors of Failure 

To investigate predictors of failed USGAVA in CIED implantation, we compared patients with successful and unsuccessful USGAVA. A failed attempt of USGAVA was more likely in patients with higher body mass index; however, unlike other USGAVA reports, such correlation resulted in a trend without reaching a statistical significance [18, 19, 23]. On the other hand, a positive relationship between successful USGAVA and AV size was shown, with a 3-fold increase of probability of success per each 1 mm increase in the AV diameter. Finally, based on the ROC curve analysis, an AV diameter ≥5.7 mm was highly accurate to predict successful USGAVA. Conversely, Ahmed and coll. showed that a successful USGAVA was significantly associated with AV depth but not AV diameter [24]. The higher mean body mass index (30.7 ± 6.4 Kg/$m^{2}$ versus 27.2 ± 4.5 Kg/$m^{2}$) and lower mean age (77 ± 10 years versus 70 ± 13 years) of their patients may provide a possible explanation for the observed discrepancy. Moreover, the substantially small numbers of unsuccessful USGAVA patients (n = 6) in our study may have determined the lack of relation with venous depth.

### 4.8 Implications in Clinical Practice 

Based on our experience, USGAVA for CIED implantation is similar in efficacy, safety, and total procedure time but significantly lower in ionizing radiation exposure compared to consolidated techniques using fluoroscopic anatomical landmarks. We reported 1.9 minutes less fluoroscopy with USGAVA than with traditional non-USGAVA access techniques. This is important in patients who may-require multiple procedures during their lifetime (e.g., device upgrade, lead revision) or for laboratory staff who perform many procedures per year. Thus, considering the guiding principle of radiation safety (ALARA principle), which states that ionizing radiations applied to humans and animals should be as low as reasonably achievable, we believe our results in minimizing radiation exposure to be worthy of emphasis. A recent study, which collected more comprehensive radiation exposure data (including Air-Kerma and Dose Area Product), showed similar results [25]. Therefore, in our current practice, USGAVA is the first-choice technique, reserving either axillary venipuncture guided by fluoroscopic landmarks or cephalic vein cutdown to the unsuccessful USGAVA cases. Though less frequently used at our Institution, cephalic vein cutdown may be preferred by the operator in selected cases (e.g., single lead pacing). Finally, contrast medium injection for venography is considered only when venipuncture guided by the fluoroscopy landmarks failed. At our Institution, USGAVA is not a routine technique in device upgrades because the operator might decide to check the patency of the venous route proximal to the AV with preprocedural venography.

### 4.9 Limitations

Some limitations have to be addressed in the present study. First, this is a single-center, retrospective, and non-randomized study; therefore, further prospective, or randomized multicenter studies are needed to confirm our results. Secondly, as a historical group of patients was used for comparison, the skills of the operators and techniques/safeguards may have improved since this historical cohort. Thirdly, as lead revisions and device upgrades have been excluded from the analysis, our results cannot be extrapolated to such procedures. Fourthly, since no mid- and long-term complications have been collected, we cannot provide safety data for comparison over a 30-day follow-up. Fifthly, while having identified a cut-off value of the AV diameter to predict successful USGAVA, we are cautious in recommending this as the sole criterion when deciding whether to proceed with USGAVA since the venous diameter is a dynamic variable whose measurement may be affected by several factors (e.g., dehydration, fluid administration). Finally, as USGAVA was performed by long-standing experienced operators in ultrasound-guided venous access in electrophysiology, our results might not be reproducible with unskilled operators.

## 5. Conclusions

The use of a HUD to guide the insertion of pacemaker or cardioverter-defibrillator leads into the axillary vein was shown to be feasible, proving similar in efficacy and safety to the traditional AV puncture guided by fluoroscopic landmarks. Furthermore, the maneuver facilitates a potential reduction in ionizing radiation exposure for both the operator and patient without lengthening the CIED implantation procedure time. Our results, in line with those of other published experiences, may facilitate the spread of the technique.
